# Concurrent light chain amyloidosis and proximal tubulopathy: Insights into different aggregation behavior—A case report

**DOI:** 10.1002/jha2.555

**Published:** 2022-09-08

**Authors:** Simone Feurstein, Julian Zoller, Constantin Schwab, Sarah Schreiner, Heiko Mundt, Iris Breitkreutz, Brigitte Schneider, Jörg Beimler, Martin Zeier, Rüdiger Waldherr, Stefan Gröschel, Carsten Müller‐Tidow, Stefan O. Schönland, Ute Hegenbart

**Affiliations:** ^1^ Department of Internal Medicine, Section of Hematology, Oncology & Rheumatology University Hospital Heidelberg Heidelberg Germany; ^2^ Institute of Pathology University Hospital Heidelberg Heidelberg Germany; ^3^ Department of Internal Medicine, Section of Nephrology University Hospital Heidelberg Heidelberg Germany; ^4^ Oncology Center Worms Worms Germany

**Keywords:** AL amyloidosis, case report, light chain proximal tubulopathy, monoclonal gammopathy of renal significance

## Abstract

Due to differences in the protein folding mechanisms, it is exceedingly rare for amyloid light chain (AL) amyloidosis and monoclonal gammopathy of renal significance (MGRS) to coexist. We herein report the first case of concurrent AL amyloidosis and a subclass of MGRS, light chain proximal tubulopathy (LCPT). The 53‐year‐old female was diagnosed with smoldering myeloma immunoglobulin G *kappa* and AL amyloidosis with deposits in fat and gastrointestinal tissue. The kidney biopsy did not show amyloid deposits but electron microscopy revealed the presence of LCPT with crystal formation in proximal tubular epithelial cells. This case illustrates the complex pathophysiology of protein deposition in monoclonal gammopathies.

1

Amyloid light chain (AL) amyloidosis, caused by misfolded antibody fragments, may damage multiple organs and organ systems with the heart, kidneys, soft tissue, liver, peripheral and autonomic nervous system being the most commonly affected [1]. Additionally, the monoclonal light or heavy chains or the underlying B‐cell clone can lead to a group of rare diseases of the kidneys, known as “monoclonal gammopathy of renal significance” (MGRS) [2]. MGRS is categorized into several different subclasses based on the type of deposit and whether the deposits are organized (e.g., light chain proximal tubulopathy [LCPT] or light chain cast nephropathy [LCCN]) or non‐organized (e.g., monoclonal immunoglobulin deposition disease [MIDD]) [2, 3, 4]. The pathogenic nature of any given light or heavy chain is strongly related to its structure, which is determined by genetic rearrangement and somatic hypermutation [5]. Due to differences in the protein folding mechanisms, it is exceedingly rare for AL amyloidosis and MIDD or LCCN to coexist, while no case of concurrent AL amyloidosis and LCPT has been described so far.

Herein, we report a 53‐year‐old female who was diagnosed with immunoglobulin G *kappa* monoclonal gammopathy of undetermined significance 3 years prior based on incidental findings. A subsequent bone marrow biopsy 5 months later showed 12% clonal plasma cells, thereby meeting criteria for smoldering myeloma, and a translocation t(11;14). Due to a mild proteinuria, a kidney biopsy was performed, which did not show any evidence of MGRS; however, electron microscopy was not performed at that time. There was no evidence of osteolytic lesions or other organ manifestations. The patient had regular follow‐up visits over the course of the next 3 years. At the latest clinic visit, a significant weight loss of 10 kg was noted. The patient also reported new onset of neuropathic pain in both hands and feet and bilateral periorbital ecchymoses. Proteinuria (0.54 g/day) and a mild decrease of the estimated glomerular filtration rate of 59.4 chronic kidney disease epidemiology collaboration were also noted. The serum‐free light chain (LC) assay revealed an elevated *kappa–lambda* ratio of 35.9 (*kappa* LCs 319 mg/L, *lambda* LCs 9 mg/L). Urine protein electrophoresis showed free *kappa*‐type Bence‐Jones proteinuria. Given the clinical suspicion for an underlying AL amyloidosis, the patient underwent a second kidney biopsy and fat aspiration. The fat aspiration showed substantial amyloid deposition on Congo red staining with positive green birefringence under polarized light (Figure 1). The kidney biopsy did not show amyloid deposits, but immunofluorescence demonstrated a stronger cytoplasmic signal for *kappa* within tubular epithelial cells. Histochemical stains and electron microscopy revealed the presence of LCPT with an increased number of lysosomes as well as rhomboid and needle‐shaped crystals in proximal tubular epithelial cells (Figure 2A and B). There was no clinical evidence of Fanconi syndrome. A retrospective examination of the initial kidney biopsy by electron microscopy also showed LCPT with intracytoplasmic crystals. A repeat bone marrow aspirate and biopsy revealed 15% clonal plasma cells and the presence of numerous Auer‐rod‐like LC crystals in the plasma cells (Figure 2C), while Congo red staining did not show any amyloid deposits. A neurological exam revealed a demyelinating sensory‐motor polyneuropathy also affecting the autonomous nervous system. Whole body magnetic resonance imaging showed no focal lesions. An upper and lower endoscopy revealed the presence of amyloid deposits and immunohistochemical staining confirmed the presence of AL *kappa* amyloid. Sequence analysis showed that the patient‐derived LC belongs to the IGKV1/D‐33 and the IGKJ3*01 family (Supporting Information, Figure S1, and Table S1). Thirteen nonsynonymous variants were identified, with five located in the complementary‐determining regions (CDRs) and eight in the framework regions.

**FIGURE 1:**
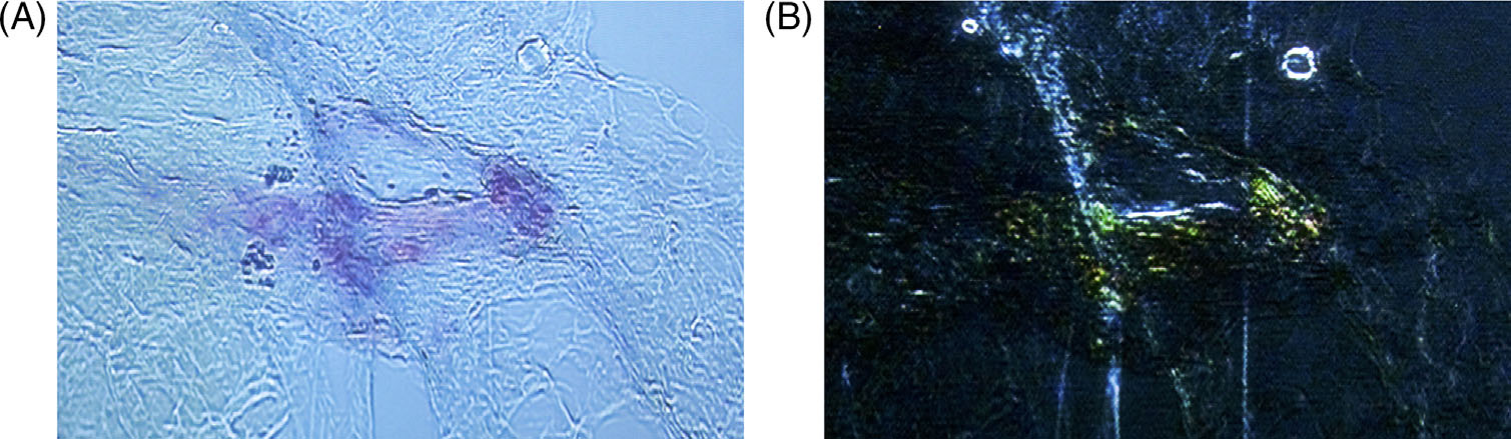
Amyloid deposits in fat tissue. Light microscopy. (A) Positive Congo red staining (red) and (B) positive green birefringence under polarized light (green).

**FIGURE 2:**
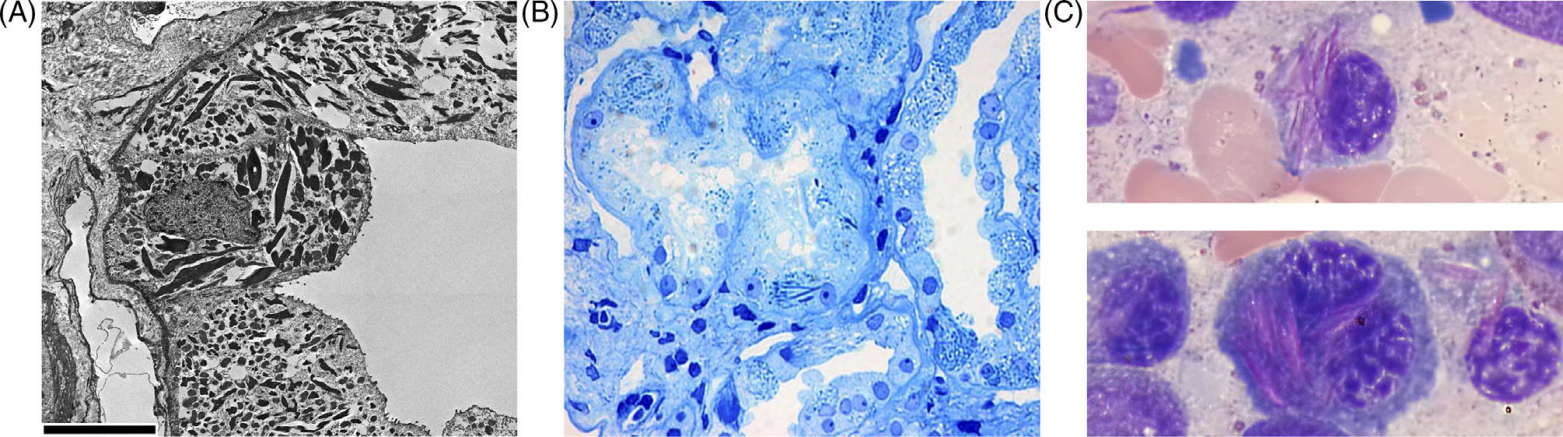
Crystalline inclusions of monoclonal *kappa* light chains in proximal tubular epithelial cells and bone marrow plasma cells. (A) Electron microscopy (bar ≙ 6 μm). Electron‐dense rhomboid and needle‐shaped crystals in proximal tubular epithelial cells. (B) Semi‐thin section of proximal tubules stained with toluidine blue. Dark blue stained crystals and granules depict intracytoplasmatic light chain deposits. (C) Light microscopy (Giemsa, oil immersion, magnification 60×). Bone marrow smear showing intracytoplasmic Auer‐rod‐like inclusions in plasma cells.

Taken together, the patient was diagnosed with systemic AL amyloidosis affecting the soft tissue as well as the peripheral and autonomic nervous and gastrointestinal system, while her proteinuria and mildly elevated creatinine were attributed to the LCPT. She received six cycles of daratumumab combined with cyclophosphamide and dexamethasone, leading to reduction of the *kappa* LCs to 135 mg/L in the serum, formerly achieving a partial remission. Her renal function and the nervous and gastrointestinal manifestations remained clinically stable. She was then switched to a combination therapy with ixazomib, lenalidomide, and dexamethasone.

Concurrent AL amyloidosis and another type of MGRS are very rare. Said et al. [6] recently reported a series of 37 patients with renal MIDD concurrent with renal AL amyloidosis with or without LCCN, which accounted for 1% of AL amyloidosis and 4% of MIDD patients, respectively. Other studies have reported single patients or small case series with renal AL amyloidosis and MIDD [4, 7, 8, 9]. Concurrent AL amyloidosis and MIDD affecting organs other than the kidneys have only been described in four patients, which included liver, bone marrow, and other organ manifestations [10, 11, 12, 13].

Some LCs induce formation of amyloid fibers, whereas others do not, making it unclear what distinguishes amyloid formers from non‐formers [2]. However, our understanding of how AL fibrils are structured has improved significantly in the last few years. Mice injected with Bence‐Jones proteins from patients with LCCN, MIDD, or AL amyloidosis developed renal lesions similar to those of the donor patients [14]. The transgenic model of MIDD demonstrated that insertion of the variable domain only of a pathogenic human LC into the mouse *kappa* locus bears the structural properties involved in its pathogenicity, and recapitulates the disease characteristics in mice [15]. Hypotheses on the coexistence of AL amyloidosis and MIDD include different conformations of the same pathogenic LC, subclonal somatic variants of the original LC gene or the presence of biclonal gammopathy. The largest study to date on combined renal AL amyloidosis and MIDD revealed that deposits had the same LC isotype and variable domain subgroup. The authors endorse the hypothesis that subclones stemming from LC rearrangements with variants in one of the CDRs may drive the phenotypic heterogeneity [6]. A similar pathomechanism may be causing the two structurally different deposits seen in our patient although other organ‐specific factors such as different pH value or chaperones may also play a role.

Our case of concurrent AL amyloidosis and LCPT illustrates the complex pathophysiology of protein deposition in monoclonal gammopathies and emphasizes that further studies examining the molecular determinants of LC aggregation propensity and proteotoxicity are needed.

## AUTHOR CONTRIBUTIONS

*Conceptualization*: Simone Feurstein, Stefan O. Schönland, and Ute Hegenbart. *Investigation*: Simone Feurstein and Ute Hegenbart. *Methodology and resources*: Julian Zoller, Constantin Schwab, Sarah Schreiner, Heiko Mundt, Iris Breitkreutz, Brigitte Schneider, Jörg Beimler, Martin Zeier, Rüdiger Waldherr, Stefan Gröschel, and Carsten Müller‐Tidow. *Visualization*: Simone Feurstein, Julian Zoller, Constantin Schwab, Brigitte Schneider, and Ute Hegenbart. *Writing—original draft*: Simone Feurstein. *Writing—review and editing*: Simone Feurstein, Ute Hegenbart, and Stefan O. Schönland. All other authors read and approved the final version.

## CONFLICT OF INTEREST

The authors declare they have no conflicts of interest.

### ETHICS STATEMENT

Written informed consent was obtained from the patient in keeping with approved institutional review board protocols and in accordance with the declaration of Helsinki.

## Supporting information

Supplement Material

## Data Availability

The data that support the findings of this study are available in the manuscript itself and the supplementary material.
