# Supplementary material for: Concurrent light chain amyloidosis and proximal tubulopathy: Insights into different aggregation behavior—A case report
Source: EJHaem. 2022 Sep 8;3(4):1377–80. doi: 10.1002/jha2.555 (PMC9713218; doi:10.1002/jha2.555)
Supplement: Supplementary file 1 — Supplement Material [file JHA2-3-1377-s001.docx]

**Supplementary material**

**Methods**

The study was approved by the local Ethics Committee of the University of Heidelberg (S-123/2006) and conducted in compliance with the declaration of Helsinki.

Sequencing of light chains

For sequence analysis, 50 ml bone marrow aspirate was collected and mononuclear cells were isolated by density gradient centrifugation (Ficoll-Paque). CD138+ plasma cell enrichment, isolation of RNA and reverse transcription was performed as previously published.^1^

Light chains (LCs) were amplified by polymerase-chain-reaction (PCR) as previously described.^2^ In brief, 1 µl template was used per 25 µl PCR reaction. A forward primer was designed to bind equally to all possible IGKV-segments and consequently allow sequencing of all possible kappa LCs. A reverse primer was designed to represent the complete IGKC-segment (Supplementary Table 1). The size and concentration of the PCR product was assessed by electrophoresis and the PCR product was subsequently purified using the ‘High Pure PCR-Product Purification Kit’ (Roche, Basel, Switzerland) according to the manufacturer’s instructions. Sanger Sequencing was performed using Eurofins GATC sequencing services.

For assignment of IGKV- and IGKJ-gene families, the VBase2 database was used.^3^ Translation of the nucleotide to an amino acid sequence was performed with the bioinformatic tool Expasy^4^ and the resulting sequence was analyzed using Ensembl BLAST.^5^ To identify patient-specific variants, the sequence was aligned with the reference sequence with the highest similarity using the multiple sequence alignment program Clustal Omega (https://www.ebi.ac.uk/Tools/msa/clustalo/). The IGKV-segment, IGKJ-segment, and IGKC-segment were aligned utilizing the Vbase2 reference (humIGKV120), the IMGT database reference^6^ (accession number J00242) and the Ensembl reference (ENSP00000374777), respectively. Based on this analysis, the patient-derived sequence could be assigned to the most prevalent IGKV-family, the IGKV1/D-33- and the IGKJ3*01-family.^7,8^ The complementarity determining regions (CDRs) and framework region (FRs) were aligned according to Kabat using abYsis.^9^ A total of 13 nonsynonymous substitutions were identified (Supplementary Figure 1). Five of them were located in the hypervariable CDRs (Q27H, N34I, N53K, D92S, N93S), while the variant T22S was located in the FR 1. Y36F and K45N were detected in the FR 2 and S65G, D70H, S76R, P80A and Y87F were found in the FR 3.

**References**

1. Berghaus N, Schreiner S, Granzow M, et al. Analysis of the complete lambda light chain germline usage in patients with AL amyloidosis and dominant heart or kidney involvement. *PLoS One*. 2022;17(2):e0264407.

2. Huhn S. ELDA qASO-PCR for High Sensitivity Detection of Tumor Cells in Bone Marrow and Peripheral Blood. *Methods Mol. Biol.* 2018;1792:1–14.

3. Retter I, Althaus HH, Münch R, Müller W. VBASE2, an integrative V gene database. *Nucleic Acids Res.* 2005;33(Database issue):D671-4.

4. Duvaud S, Gabella C, Lisacek F, et al. Expasy, the Swiss Bioinformatics Resource Portal, as designed by its users. *Nucleic Acids Res.* 2021;49(W1):W216–W227.

5. Howe KL, Achuthan P, Allen J, et al. Ensembl 2021. *Nucleic Acids Res.* 2021;49(D1):D884–D891.

6. Giudicelli V, Chaume D, Lefranc M-P. IMGT/GENE-DB: a comprehensive database for human and mouse immunoglobulin and T cell receptor genes. *Nucleic Acids Res.* 2005;33(Database issue):D256-61.

7. Abraham RS, Geyer SM, Price-Troska TL, et al. Immunoglobulin light chain variable (V) region genes influence clinical presentation and outcome in light chain-associated amyloidosis (AL). *Blood*. 2003;101(10):3801–8.

8. Kourelis T V., Dasari S, Theis JD, et al. Clarifying immunoglobulin gene usage in systemic and localized immunoglobulin light-chain amyloidosis by mass spectrometry. *Blood*. 2017;129(3):299–306.

9. Swindells MB, Porter CT, Couch M, et al. abYsis: Integrated Antibody Sequence and Structure—Management, Analysis, and Prediction. *J. Mol. Biol.* 2017;429(3):356–364.

**Supplementary Figures and Tables**

**Supplementary Figure 1**

1 **FR1**   **CDR1** 38
P3964.21 ---------------------------QSPSSLSASVGDRVTISCQASHDISNYLIWFQQ

IGKV1/D-33_Vbase2 ----------------------DIQMTQSPSSLSASVGDRVTITCQASQDISNYLNWYQQ

IGKV1/D-33-201_Ensembl MDMRVPAQLLGLLLLWLSGARCDIQMTQSPSSLSASVGDRVTITCQASQDISNYLNWYQQ

IGKJ3*01_IMGT ------------------------------------------------------------

IGKC-201_Ensembl ------------------------------------------------------------

**FR2** **CDR2 FR3**   **CDR3** 98

P3964.21 KPGKAPNLLIYDASKLETGVPSRFSGGGSGTHFTFTIRSLQAEDIATYFCQQYSSLPFTF

IGKV1/D-33_Vbase2 KPGKAPKLLIYDASNLETGVPSRFSGSGSGTDFTFTISSLQPEDIATYYCQQYDNL----

IGKV1/D-33-201_Ensembl KPGKAPKLLIYDASNLETGVPSRFSGSGSGTDFTFTISSLQPEDIATYYCQQYDNLP---

IGKJ3*01_IMGT ---------------------------------------------------------FTF

IGKC-201_Ensembl ------------------------------------------------------------

158

P3964.21 GPGTKVDIKRTVAAPSVFIFPPSDEQLKSGTASVVCLLNNFYPREAKVQWKVDNALQSGN

IGKV1/D-33_Vbase2 ------------------------------------------------------------

IGKV1/D-33-201_Ensembl ------------------------------------------------------------

IGKJ3*01_IMGT GPGTKVDIK---------------------------------------------------

IGKC-201_Ensembl ---------XTVAAPSVFIFPPSDEQLKSGTASVVCLLNNFYPREAKVQWKVDNALQSGN

211

P3964.21 SQESVTEQDSKDSTYSLSSTLTLSKADYEKHKVYACEVTHQGLSSPVTKSFNR---

IGKV1/D-33_Vbase2 --------------------------------------------------------

IGKV1/D-33-201_Ensembl --------------------------------------------------------

IGKJ3*01_IMGT --------------------------------------------------------

IGKC-201_Ensembl SQESVTEQDSKDSTYSLSSTLTLSKADYEKHKVYACEVTHQGLSSPVTKSFNRGEC

**Supplementary Figure 1. Alignment of the full-length light chain sequence compared to the respective reference sequences.** Highlighted in yellow are nonsynonymous substitutions. Complementarity-determining regions are underlined. The amino acid sequence was numbered based on the Vbase2 reference.

**Supplementary Table 1. Primer used for Sanger Sequencing.**

| **Name** | **Primer sequence** |
| --- | --- |
| VKKL_A_fw | CCAGATGACCCAGTCTCC |
| CKKL_A_rv | CACTCTCCCCTGTTGAAGC |
